# Lsh/HELLS regulates self-renewal/proliferation of neural stem/progenitor cells

**DOI:** 10.1038/s41598-017-00804-6

**Published:** 2017-04-25

**Authors:** Yixing Han, Jianke Ren, Eunice Lee, Xiaoping Xu, Weishi Yu, Kathrin Muegge

**Affiliations:** 1 Mouse Cancer Genetics Program, Center for Cancer Research, National Cancer Institute, Frederick, Maryland 21702 USA; 2 Basic Science Program, Leidos Biomedical Research, Inc., Mouse Cancer Genetics Program, Frederick National Laboratory for Cancer Research, Frederick, Maryland 21702 USA

## Abstract

Epigenetic mechanisms are known to exert control over gene expression and determine cell fate. Genetic mutations in epigenetic regulators are responsible for several neurologic disorders. Mutations of the chromatin remodeling protein Lsh/HELLS can cause the human Immunodeficiency, Centromere instability and Facial anomalies (ICF) syndrome, which is associated with neurologic deficiencies. We report here a critical role for Lsh in murine neural development. Lsh depleted neural stem/progenitor cells (NSPCs) display reduced growth, increases in apoptosis and impaired ability of self-renewal. RNA-seq analysis demonstrates differential gene expression in *Lsh−/−* NSPCs and suggests multiple aberrant pathways. Concentrating on specific genomic targets, we show that ablation of Lsh alters epigenetic states at specific enhancer regions of the key cell cycle regulator Cdkn1a and the stem cell regulator Bmp4 in NSPCs and alters their expression. These results suggest that Lsh exerts epigenetic regulation at key regulators of neural stem cell fate ensuring adequate NSPCs self-renewal and maintenance during development.

## Introduction

During embryogenesis, the cerebral cortex develops from multipotent neural stem cells that begin as neuroepithelial cells in the ventricular zone (VZ) and expand into the intermediate neural progenitors in the subventricular zone (SVZ)^1^. A neural stem cell is capable of self-renewal (by symmetric division) for extended periods of time. Asymmetric cell division allows neural stem cells to generate another stem cell and a progenitor cell (immature and proliferating cells) that is capable to differentiate into distinct neural lineages^2, 3^. Thus, neural stem/progenitor cells (NSPCs) can serve as a tool to study neural development.

Instructions for self-renewal and differentiation, two defining features of neural stem cells, are regulated by intrinsic and extrinsic signals from the neurogenic niche^4, 5^. Epigenetic regulation plays a pivotal role in the maintenance of cell identity as well as the stepwise guidance towards cellular differentiation. Chromatin states of NSPCs change gradually during this process^6^. Genetic mutations of epigenetic modifiers are responsible for human diseases some of which have neurologic deficiencies^7^. Understanding molecular mechanisms and identifying key chromatin factors that regulate neural stem cell maintenance and neurogenesis is critical for understanding normal neural development, to learn about neurological disorders and to discover molecular pathways that could be targeted for therapy^8, 9^.

Epigenetic changes during neural development comprise alterations in histone modifications and DNA methylation. In particular, genetic mutations that are involved in setting, removing and reading DNA methylation patterns are known to cause neurologic defects. For example, genetic mutation of the methyl-DNA binding protein MECP2 leads to the Rett syndrome^10^ and genetic mutations causing DNA hypomethylation result in the ICF (immunodeficiency, centromeric instability, facial anomalies) syndrome^11, 12^. The ICF syndrome is a severe disease that often leads to lethality at a young age; the hallmark of the disease is a severe immunodeficiency with varying degrees of facial dysmorphism, and neurologic defects. Four genes have been identified that upon genetic mutations cause the ICF syndrome, among them are the DNA methyltransferase DNMT3B and the chromatin remodeling protein Lsh (also known as HELLS)^13^. Patients with genetic mutation of HELLS show a delay in the development of motor skills and evidence of mental retardation^14^. However, the reason for these neurologic deficiencies remains unknown.

Murine Lsh shares 95% protein homology with human HELLS^15^. The deletion of two critical domains of Lsh, the ATP binding site and the DEAD box^16^, is lethal in mice and leads to severe defects including kidney necrosis, deficiencies in hematopoietic stem cells, and defects in the male and female germ cell^17–20^. The deletion of two other conserved domains in mice, yields reduced, but detectable small amounts of Lsh protein, and results in a less severe phenotype with early death around weaning and an aging phenotype^21^. Lsh modulates DNA methylation patterns in mice^22–26^ and fibroblasts derived from *Lsh−/−* embryos show a 40% reduction of CG methylation compared to wild type cells^27^. In addition, somatic tissues, such as embryonic brain, show a dramatic reduction of cytosine methylation indicating an epigenetic function of Lsh in the nervous system^28^. *Lsh−/−* embryos cannot survive beyond birth and thus an assessment of neurologic function and motor neuron delay has been impossible. It is currently unknown whether Lsh mediated chromatin changes affect neural stem cell renewal or influence neural differentiation pathways into distinct lineages. Here, we examined NSPCs derived from *Lsh−/−* embryos and determined chromatin states at specific genomic loci, assessed gene expression, and the capacity for cellular differentiation, proliferation and stem cell renewal.

## Results

### Reduced self-renewal in *Lsh−/−* neurospheres

During embryonic development high levels of mRNA are detectable in the developing brain^29^, albeit expression is not exclusive for a specific stage or tissue type and is associated with cellular proliferation^15, 21, 29^. Genomic DNA derived from brain tissue of E18.5 gestation *Lsh−/−* embryos shows greater than 30% reduction of cytosine methylation compared to wild type (WT) controls^27, 28^, indicating a role for Lsh as epigenetic regulator in neural cells.

**Figure 1:**
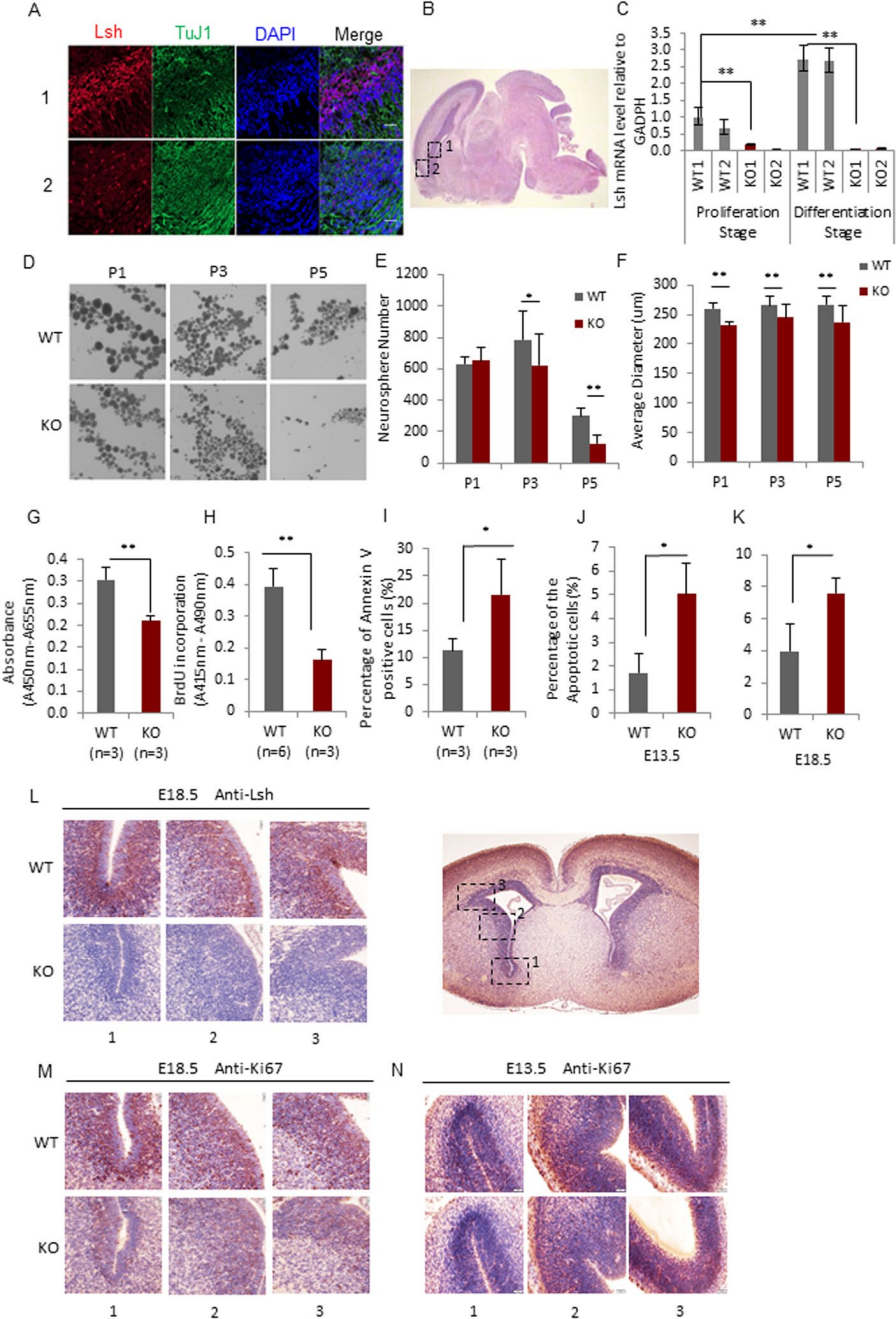
Lsh is required for proliferation and self-renewal of NSPCs. (**A**) Immunofluorescence for detection of Lsh and Tuj1 in E18.5 (day 18.5 gestation) embryonic brain sections. Magnificence: 40x, Scale bar, 20 μm. (**B**) A representative sagittal section of the embryonic brain, the number labeled areas are shown in higher magnification in (**A**). (**C**) Lsh mRNA evaluation by RT-qPCR during NSPCs proliferation and differentiation *in vitro*. Gapdh served as internal control, expression levels were relative to WT1 at the proliferation stage. (**D**) Representative phase contrast images for neurospheres at passage 1, 3 and 5. (**E** and **F**). Quantification analysis of neurosphere numbers (**E**) and the average diameter (**F**) in WT and Lsh KO NSPCs at passage1, 3 and 5 (3 WT and 3 KO embryos were used, 5 technical repeats for each sample). (**G**) Cell viability assay by WST-1 in NSPCs cultures. (**H**) BrdU incorporation assay in NSPCs cultures. (**I**) Apoptotic cell measurement in NSPCs cultures by FACS. (**J** and **K**) Quantification of TUNEL assay on E13.5 (**J**) and E18.5 (**K**) brain tissue (SVZ) sections. Multiple SVZ areas were quantified by Image J (n = 4). (**L**,**M** and **N**) IHC by anti-Lsh and anti-Ki67 on E18.5 embryonic brain sections (n = 4). A representative transverse section of the embryonic brain is shown (4x), the number labeled areas are shown in higher magnification (40x) in (**L**,**M**,**N**). Brown signal indicates positive protein stains and blue represents the DAPI signals. Scale bar, 20 μm. *p < 0.05, **p < 0.001. Error bars represent SD.

To determine the expression of Lsh in the brain we used immunofluorescence staining. Lsh protein expression was readily detectable in embryonic brain tissue sections (Fig. 1A,B). The cortex region (Fig. 1B region 2) exhibited only a few positive cells, while more Lsh staining was observed in the proliferating zones lining the ventricles, including the SVZ (Fig. 1B region 1) enriched for neural progenitors, and the VZ with some scattered Lsh expressing cells (Fig. 1A,B,L). Furthermore, Lsh mRNA was expressed in neurosphere cultures that are enriched for NSPCs (Fig. 1C). The presence of Lsh in neurosphere cultures suggests that Lsh is expressed from an early stage of neurogenesis.

*Lsh−/−* mice die at birth and newborn brain does not reveal histologic anomalies^30^. To determine whether Lsh plays a functional role in NSPCs, we examined the ability for self-renewal/proliferation and differentiation, two key characteristics of stem cells. VZ and SVZ of the lateral ventricle wall harbor NSPCs^31^. When dissociated cells are cultured *in vitro* in the presence of basic fibroblast growth factor (bFGF) and epidermal growth factor (EGF)^32^, neural stem cells and their progeny grow as non-adherent cell clusters, termed neurospheres^33–35^. The number of clonal outgrowth in a ‘neurosphere assay’ serves as assessment for the self-renewing capacity of stem cells^32, 36^. Using the neurosphere assay, we found that the number of primary neurospheres was similar comparing WT to *Lsh−/−* cultures, but the size of *Lsh−/−* neurospheres was significantly reduced (Fig. 1D,E and F) which may reflect a reduced rate of cell division of self-renewing stem cells and/or their progeny. Next, we explored the self-renewal ability of neural stem cells. Upon dissociation of neurospheres, neural stem cells give rise to new sphere colonies at a frequency of 19% to 44% (for primary and secondary neurospheres, respectively)^37^. When we repeatedly dissociated and re-plated neurospheres at clonal density, we observed a 60% reduction of *Lsh−/−* neurosphere numbers (P5) (Fig. 1D,E) and a decrease in the size of *Lsh−/−* neurospheres (Fig. 1F) suggesting impaired self-renewal and proliferation of NSPCs. Measuring mitochondrial dehydrogenase (WST-1) activity, we found that *Lsh−/−* cells had a 30% decrease compared to WT cells indicating reduced viability and proliferation (Fig. 1G). Furthermore, incorporation of BrdU, a synthetic nucleoside analog that is incorporated into DNA during replication, was significantly decreased by about 60% in *Lsh−/−* cells (Fig. 1H). Staining with annexin V, a measure for apoptosis, was about two fold increased in *Lsh−/−* cells (Fig. 1I). This data suggests that the impaired growth capacity of *Lsh−/−* NSPCs *in vitro* is due, in part, to impaired replication and increased apoptosis.

To assess asymmetric cell division capacity of neural stem cells, we monitored the asymmetric cell division maker distribution of Numb (Supplement Fig. 1A,B) and Stau2 (not shown) using time-lapse microscope that allows tracking of daughter cells^38–40^. The frequency of asymmetric distribution of Numb and Stau2 was indistinguishable comparing *Lsh−/−* cell pairs with WT pairs (Supplement Fig. 1C) providing no evidence that Lsh depletion alters asymmetric cell division.

To investigate the effect of Lsh deletion on NSPCs *in vivo*, we performed immunohistochemistry (IHC) on E18.5 murine brain sections for detection of Lsh and the proliferation cellular marker Ki67. The majority of WT cells lining the ventricle, including the SVZ, stained positive for Ki67, indicating active proliferation (Fig. 1M). Since the majority of these cells stained positive for Lsh (Fig. 1L), we conclude that actively proliferating cells of the SVZ co-express Lsh. In contrast to WT sections, *Lsh−/−* brain sections showed a decrease of Ki67 positive cells indicating reduced proliferation *in vivo*. A similar reduction of Ki67 expression changes was observed at day 13.5 of gestation (Fig. 1N). Additionally, the proportion of apoptotic cells *in vivo* visualized by the TUNEL assay was increased from 1.7 to 5% and from 3.9 to 7.6% in *Lsh−/−* E13.5 and E18.5 brain sections, respectively (Fig. 1J,K). Altogether, the observation of reduced proliferation/survival in *Lsh−/−* embryos *in vivo* is consistent with the repressed neurosphere assay *in vitro*, suggesting Lsh is required in the self-renewal and proliferation capacity of NSPCs.

### Delayed linage commitment in the absence of Lsh

**Figure 2:**
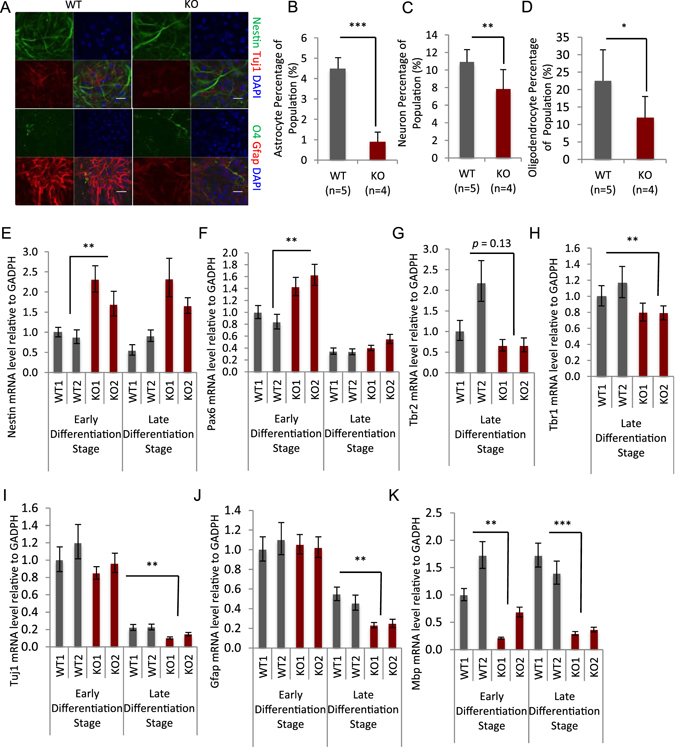
Delay of neural linage commitment in *Lsh−/−* NSPCs. (**A**) Immunofluorescence assay using neural linage markers in differentiated NSPCs *in vitro* using an undirected differentiation protocol. Magnification: 40x, Scale bar, 20 μm. (**B**,**C** and **D**) Quantification of mature linages using lineage-oriented differentiation protocols through which NSPCs differentiated into one linage dominant populations: astrocyte (**B**), neuron (**C**) and oligodendrocyte (**D**), proportion measurement by FACS. See also Fig. S2. (**E** and **F**) mRNA level assessment of NSPCs progenitor markers by RT-qPCR: Nestin (**E**) and Pax6 (**F**). (**G** and **H**) mRNA level assessment of markers by RT-qPCR: Tbr2 (**G**) and Tbr1 (**H**). (**I**,**J** and **K**) mRNA level assessment of mature neural linage markers by RT-qPCR: Tuj1 (**I**) for neuron, Gfap (**J**) for astrocyte and Mbp (**K**) for oligodendrocyte. Early and late differentiation stage refers to differentiation for 3 and 6 days, respectively. Gapdh served as internal control, expression levels were relative to WT1 for each gene. Data are represented as mean ± SD. *p < 0.1, **p < 0.05, ***p < 0.001.

To address the question whether the repression of neurosphere growth may be due to premature differentiation, we investigated the differentiation ability of neural stem cells. Using an undirected differentiation protocol we readily detected the expression of lineage specific markers Tuj1 (a marker for﻿ neuron), Gfap (a marker for astrocytes) and O4 (a marker of immature pre-myelinating oligodendrocytes) in WT and *Lsh−/−* cultures (Fig. 2A), albeit we found that the expression of neuron and astrocyte markers was slightly reduced in *Lsh−/−* cultures. For further quantification, *in vitro* lineage oriented differentiation protocols were applied. After one﻿ of the dominant neural linage population derived, we employed FACS analysis and confirmed that *Lsh−/−* stem cells were capable to express different neural specific lineage markers (Fig. 2B,C and D). Furthermore, none of the conditions yielded increased lineage marker expression in *Lsh−/−* cultures, suggesting that premature differentiation is not responsible for impaired growth of *Lsh−/−* neurospheres. On the contrary, expression of Gfap^+^Nestin^−^ was significantly decreased by about 80% compared to WT controls, Tuj^+^Nestin^−^ expression was reduced by 30%, and O4^+^Tbr2^−^ was decreased by almost 50%, suggesting impaired or delayed lineage specific cell commitment in the absence of Lsh. This data is consistent with a transitional block in the progression towards mature cell types in the absence of Lsh.

To assess a potential delay in maturation, we monitored mRNA expression for key progenitor and differentiation markers using qPCR assay at early (differentiation for 3 days) and late (differentiation for 6 days) stages. The progenitor markers Nestin and Pax6 were not adequately downregulated during differentiation in *Lsh−/−* cultures compared to WT controls (Fig. 2E,F). This was most notable at an early stage of differentiation (day 3). In contrast, Tbr2, an intermediate marker^41^ (Fig. 2G), Tbr1, a postmitotic marker expressed at a later stages^41, 42^ (Fig. 2H) and the lineage markers (Tuj1, Gfap and Mbp) were significantly reduced in *Lsh−/−* cultures at day 6, consistent with a maturation delay in *Lsh−/−* cultures (Fig. 2I,J and K). In addition, WT cultures expressed small amounts of lineage markers (Tuj1, Gfap, Mbp) in the absence of exogenous differentiation signals^43^, whereas no mature lineage markers were expressed in *Lsh−/−* neurospheres under these conditions (Supplement Fig. 2A,B).

Collectively, our data suggests that *Lsh−/−* neural stem cells are capable to develop into the main neural lineages, albeit with a slight delay of mature marker expression under *in vitro* conditions. We conclude that the impaired capacity for proliferation/self-renewal in the absence of Lsh is not due to premature differentiation but involves a distinct molecular pathway.

### Altered Bmp4 and Cdkn1a expression in *Lsh−/−* NSPCs

**Figure 3:**
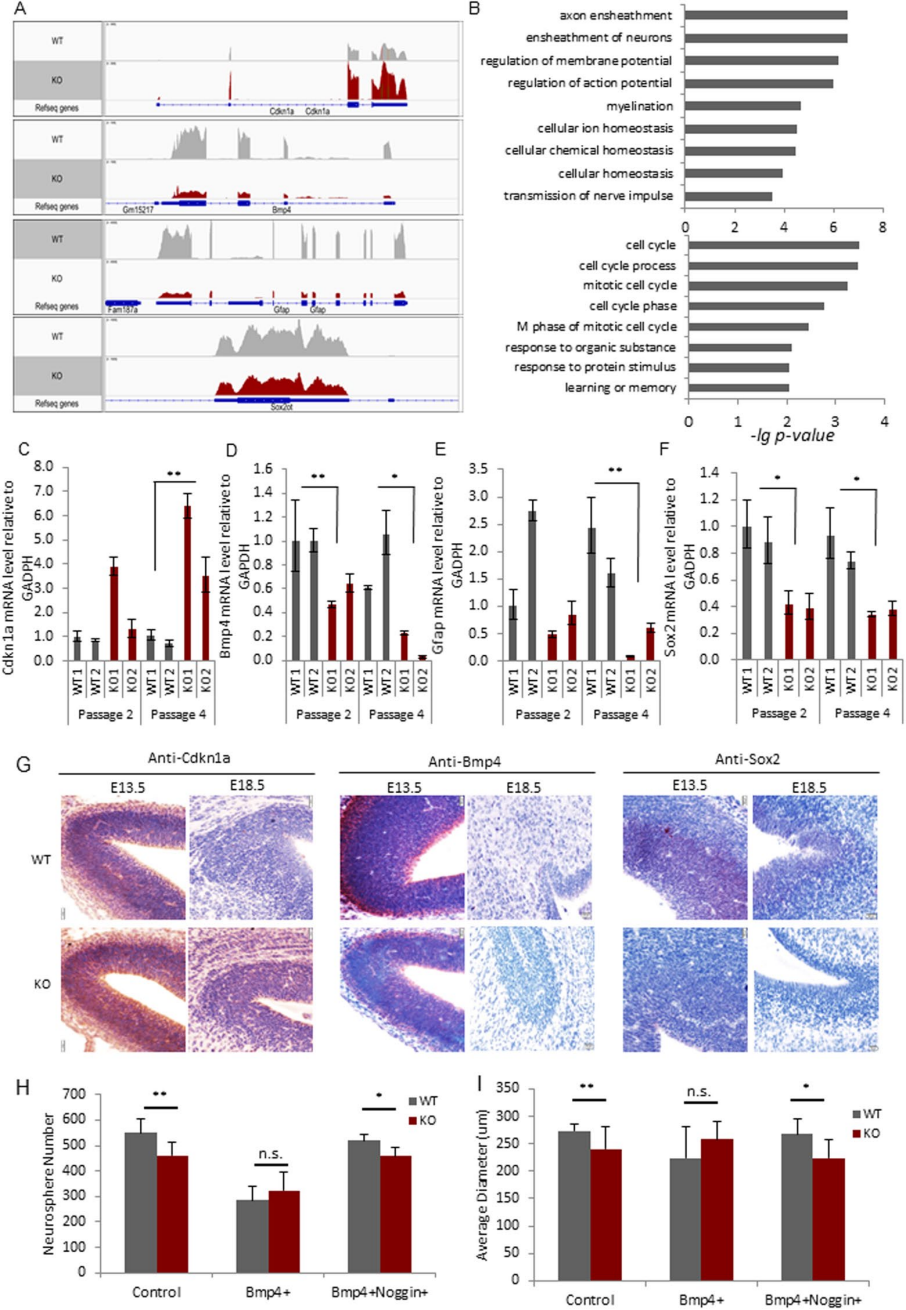
Altered Bmp4 and Cdkn1a expression in the absence of Lsh. (**A**) Profiles of RNA-seq reads at specific genomic regions: Cdkn1a, Bmp4, Gfap and Sox2 (the Sox2ot transcript (chr3: 34,459,303–34,576,915) encompasses the Sox2 transcript (chr3: 34,548,927–34,551,382) –the graph shows the read numbers of the Sox2 gene and the coding region is represented in blue boxes at the top of the figure) (**B**). Gene Ontology (GO) analysis of genes down-regulated (upper panel) or up-regulated (lower panel) in Lsh*−/−* NSCs. X-axis represents negative lg *p*-values. See also Fig. S3. (**C**,**D**,**E** and **F**) Validation of gene expression changes by RT-qPCR: Cdkn1a (**C**), Bmp4 (**D**), Gfap (**E**) and Sox2 (**F**). (**G**) IHC assay on different development stage embryonic brain sections (n = 4) using anti-Bmp4 (E13.5), anti-Sox2 (E13.5) and anti-Cdkn1a (E13.5 and E18.5) antibodies. (**H** and **I**) Quantification analysis of neurosphere numbers (**H**) and average diameters (**I**) in WT and KO NSPCs upon addition of exogenous Bmp4. Noggin was used as antagonist of Bmp4 to reverse Bmp4 effects. (3 WT and 3 KO embryos were used, 3 technical repeats for each sample). See also Fig. S2. Gapdh served as internal control, expression levels were relative to WT1 for each gene. Error bars represent SD. Scale bar, 20 μm. *p < 0.05, **p < 0.001.

To reveal key factors that mediate growth repression upon Lsh deletion, we used an unbiased approach and performed RNA-seq analysis in passage 2 neurospheres. We found 106 differentially expressed genes comparing *Lsh−/−* to WT samples (FDR < 0.05) (Fig. 3A,B, Supplement Table 1). Gene ontology analysis indicated that differentially expressed genes act in the regulation of transcription, cell division and neuron development (Fig. 3B, Supplement Fig. 3). Consistent with our previous analysis, the neural lineage markers, Mbp and Gfap were significantly decreased. On the other hand, developmental factors such as Egr1 and Egr2, two transcription factors that upon mutation lead to polyneuropathy and memory dysfunction^44, 45^, were significantly upregulated (Supplement Table 1) indicating abnormal gene expression in the absence of Lsh. Markedly, Cdkn1a was significantly increased upon deletion of Lsh, whereas Bmp4 was significantly reduced (Fig. 3A). Cdnk1a is a cell cycle inhibitor that represses Sox2 expression and reduces the number of neural stem cells^46, 47^. We also noted a slight reduction in Sox2 read numbers (1.6 fold) in Lsh*−/−* samples (though not significant by stringent RNA-seq analysis) (Fig. 3A, Supplement Table 2). Bmp4 is a member of the family of bone morphogenic growth and proliferation factors which has been implied in neurogenesis^48^. The differential expression of Bmp4, Cdkn1a, Gfap and Sox2 was further validated in neurosphere cultures derived from different embryos using RT-qPCR analysis (Fig. 3C,D,E,F). Furthermore, IHC on embryonic brain sections was performed to examine expression of the growth regulatory factors Cdkn1a and Bmp4 *in vivo*. Consistent with *in vitro* data, we observed increased Cdkn1a protein level in embryonic E13.5 and E18.5 brain sections, whereas Bmp4 protein expression was reduced in *Lsh−/−* samples compared to WT controls (Fig. 3G). In addition, we found a slight decrease of the progenitor marker Sox2 in the absence of Lsh (Fig. 3G). The reduced mRNA and Sox2 protein level are consistent with the reported repressor function of Cdkn1a on Sox2^47^ and the reduced expansion of neural progenitors in the absence of Lsh.

Since the ICF syndrome shows genome instability, we considered the possibility that activation of a repair pathway may contribute to p21 (Cdkn1a) increases via p53 activation. However, we could not detect increases of γH2AX (data not shown) or any evidence of p53 mRNA or protein elevation in the absence of Lsh (Supplement Fig. 2C,D). Furthermore, phosphorylation of p53 was not detectable in eitherwild type or *Lsh−/−* neuropsheres (but was present in MMS treated ES cells, serving as positive control) (Supplement Fig. 2D). While we cannot exclude a DNA damage induced response pathway, we did not find any evidence of genomic instability and p53 activation in *Lsh−/−* neurosphere cultures.

Since Bmp4 regulates neural stem cell quiescence^49^, we addressed its role in the neurosphere self-renewal assay. Consistent with our previous observation (Fig. 1D,E and F), clonal expansion was significantly decreased in *Lsh−/−* neurospheres compared to WT cultures (Fig. 3H,I). Noggin, an inhibitor of Bmp4 could slightly reduce the size and number of WT neurospheres in culture (Supplement Fig. 4C,D) suggesting a role for Bmp4 in proliferation and/or protection against apoptosis. The partial reduction by Noggin (which was not at *Lsh−/−* levels) may suggest an incomplete inhibition or that other Lsh effects, in addition to Bmp4, influence the growth. A moderate level of Bmp4 addition had a differential effect on WT versus *Lsh−/−* cultures displaying a more pronounced reduction of neurosphere numbers in WT culture (50% reduction) compared to *Lsh−/−* culture (30% reduction). It should be noted that the repressive effect of recombinant Bmp4 has been previously observed^50^ and has been attributed to an antagonistic inhibitory effect of extrinsic Bmp4 to FGF2 responsiveness^51^. Furthermore, we found that addition of exogenous Bmp4 reduced *Cdkn1a* mRNA level in *Lsh−/−* cultures, supporting a differential role of exogenous Bmp4 on *Lsh−/−* NSPCs compared to controls (Supplement Fig. 5). The differential effect of growth suppression and *Cdkn1a* mRNA suggests that responsiveness of NSPCs to Bmp4 is modulated by Lsh deletion.

### Epigenetic changes at enhancers of Bmp4 and Cdkn1a in *Lsh−/−* NSPCs

Lsh belongs to a family of chromatin remodeling factors and is critical for the establishment of DNA methylation patterns during development^22, 24, 52^. Lsh can directly alter accessibility to DNA sequences, in part, by altering nucleosome density at specific genomic regions^16^. Genome wide analysis has demonstrated widespread, but locus specific changes in DNA methylation and altered histone modifications in dependence of Lsh^17^.

**Figure 4:**
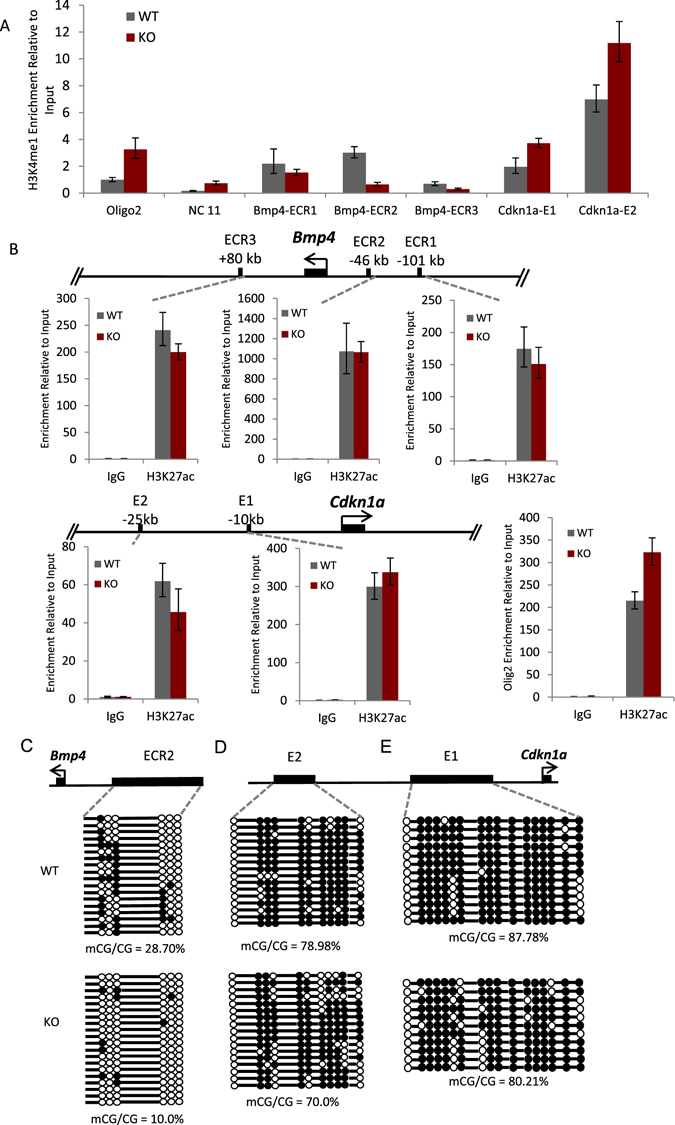
Altered epigenetic marks at *Bmp4* and *Cdkn1a* enhancer regions. (**A** and **B**) (**A**) ChIP-qPCR assay for detection of histone marker H3K4me1 at Bmp4 enhancer regions and Cdkn1a enhancer regions as depicted in (**B**). Oligo2 and NC 11 represent positive and negative controls. (**B**) ChIP-qPCR assay for detection of histone marker H3K27ac at Bmp4 and Cdkn1a enhancer regions. Oligo2 served as positive control and Ig was used as negative control. (**C**,**D** and **E**) CpG methylation assessment by bisulfate sequencing assay on enhancer regions of Bmp4 (**C**) and enhancer regions of Cdkn1a (**D** and **E**). White circles represent unmethylated CpG sites, and black circles represent methylated CpG sites. WT and KO samples for BS assay n = 2. A similar reduction in CG methylation was observed in biologic repeats (Supplement Fig. 7A,B).

To characterize further the molecular mechanisms by which Lsh modulates the expression of the key regulators Bmp4 and Cdkn1a, we initially performed a ChIP assay to determine Lsh occupancy. Using chromatin derived from neurospheres (passage 2) we examined the *Bmp4* regulatory region, which contains three enhancer regions (ECR, evolutionarily conserved regions)^53^ and the Cdkn1a regulatory region with two regulatory sites^50^. Initially, we established the presence of Lsh at those enhancer sites (Supplement Fig. 6). This is consistent with previous genome-wide analysis, which has demonstrated a preferential occupancy of Lsh or other chromatin remodelers at regulatory sites/promoter regions^54, 55^. However, the effect of Lsh on DNA methylation is widespread (about 30% reduction) suggesting a broad distribution of Lsh occupancy in the genome. Furthermore, the presence of chromatin remodeling factors is not predictive for their action and only a deletion or mutation can demonstrate their function^55^. Examining H3K4me1, which designate enhancers, we observed a 30% and 60% reduction at enhancer 1 (ECR1) and enhancer 3 (ECR3), respectively (Fig. 4A). The most dramatic change was observed at enhancer 2 (ECR2), where the level of H3K4me1 was reduced more than 4-fold in *Lsh−/−* neurospheres compared to WT cultures. In contrast, H3K4me1 levels were 1.9-fold and 1.6-fold increased at the essential enhancer 1 and 2 of the *Cdkn1a* gene^56^ in *Lsh−/−* samples compared to WT cultures (Fig. 4B). In addition, H3K27ac, a frequent mark at functional enhancer, was present at all examined loci and was comparable to the H3K27ac level at the active Olig2 promoter regions which served as positive control) (Fig. 4B). These results indicate the presence of Lsh at several regulatory regions, such as the *Bmp4* enhancer 2 region.

Since Lsh mediates its effects in part via DNA methylation changes, we next examined CG methylation in neurosphere cultures by employing bisulfite-sequencing analysis. The enhancer 2 of the *Bmp4* gene exhibits an approximate three-fold reduction in DNA methylation levels comparing *Lsh−/−* samples to WT controls (28.7% versus 10%, respectively) (Fig. 4C, Supplement Fig. 7A). In contrast, enhancer 1 and enhancer 2 of the *Cdkn1a* gene revealed no significant changes in DNA methylation in the absence of Lsh, but exhibited a trend of slight CG methylation reduction in *Lsh−/−* samples (Fig. 4D,E, Supplement Fig. 7B).

**Figure 5:**
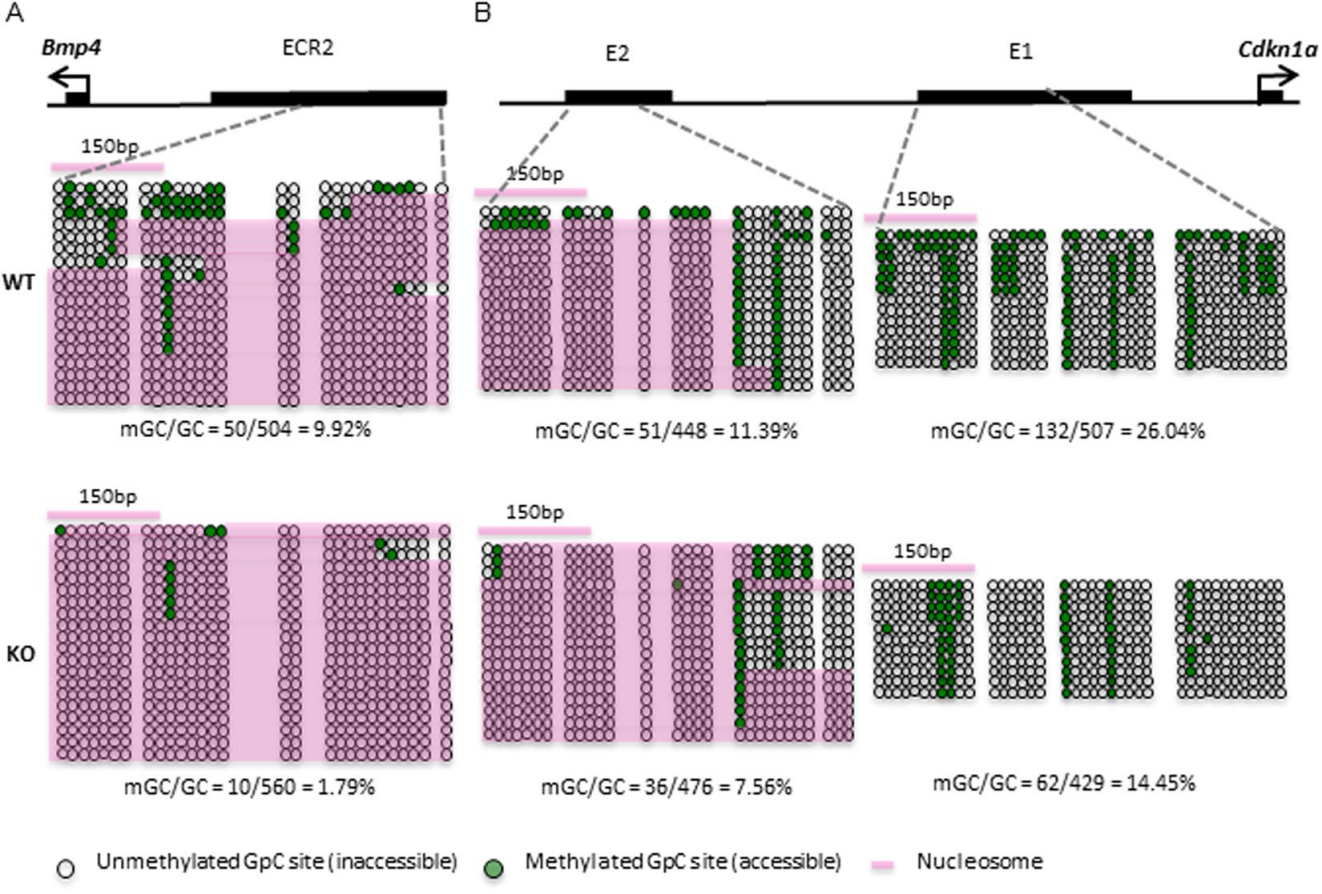
Altered nucleosome occupancy at the regulatory regions of *Bmp4* and *Cdkn1a*. GpC methylation and nucleosome position assay by NOME-seq at Bmp4 enhancer region (**A**) and Cdkn1a enhancer regions (**B**). WT and KO samples for NOMe-seq assay n = 2. A similar change in nucleosome occupancy was observed in a biologic repeat (Supplement Fig. 8).

Finally, we examined directly chromatin accessibility in *Lsh−/−* neurospheres using the NOMe-seq assay. Previous results have demonstrated that Lsh deletion alters nucleosome occupancy and chromatin accessibility at repeat sequences and that this activity depends on chromatin remodeling function of Lsh^16^. The enhancer 2 of the *Bmp4* gene showed a reduction of chromatin accessibility in *Lsh−/−* samples compared to WT controls (Fig. 5A, Supplement Fig. 8A). In particular, an about 100 bp region at the 5′end of ECR2 region suggested an absence of nucleosomes in 7 out of 18 alleles in KO samples (biologic repeat 7/14) compared to 90 to 100% coverage in controls. 7/18 alleles may present 39% of the cell population (with a homogenous state at both alleles), or may present 78% (with heterogeneous alleles). This accessibility change is consistent with the decrease of H3K4me1 (Fig. 4A) and suppression of Bmp4 mRNA (Fig. 3D) in *Lsh−/−* samples. Enhancer 1 and enhancer 2 of the *Cdkn1a* gene did not show overall consistent changes in chromatin accessibility comparing *Lsh−/−* to WT samples (Fig. 5B, Supplement Fig. 8B).

Altogether, differential chromatin accessibility, CpG methylation, and active chromatin marks were identified in the absence of Lsh. These results suggest that the regulation of Bmp4 under the control of Lsh in NSPCs is based on epigenetic mechanisms, most prominently at regulatory regions of the *Bmp4* gene.

## Discussion

The chromatin remodeling protein Lsh is critical for survival of mice and controls genome wide DNA methylation level in several tissues, including the brain. Here, we demonstrate that Lsh deletion affects self-renewal/growth and apoptosis of NSPCs and leads to deregulated expression of Bmp4 and the cell cycle inhibitor Cdkn1a. We also report that Lsh deletion modulates responsiveness to Bmp4 with respect to growth repression and Cdkn1a mRNA regulation. Specific chromatin changes at the Bmp4 enhancer region, including altered nucleosome density, DNA methylation and H3K4me1 modifications are induced by Lsh deletion and associated with reduced levels of Bmp4 mRNA. Our data suggests a hierarchy of Lsh effects, from chromatin induced changes to de-regulation of stem cell regulators and cell cycle effectors and impaired self-renewal capacity of NSPCs.

Lsh is a chromatin remodeling protein, and its ability to alter nucleosome occupancy may be, in part, responsible for the loss of DNA methylation level in Lsh deficient tissues^16^. Here, we report that Lsh not only exerts its chromatin remodeling activity on repeat sequences^16^, but we also demonstrate chromatin changes at the Bmp4 enhancer site. It should be noted that the NOMe-seq assay, similar to other methods of profiling chromatin accessibility^57^, may be influenced by DNA binding factors. However, the NOMe-seq assay identifies most promoter and enhancer regions as chromatin accessible or nucleosome depleted^58, 59^.

While the presence of Lsh at repeats reduces chromatin accessibility, we observed here improved chromatin access at the Bmp4 enhancer region when Lsh in present. Chromatin remodeling activities comprise nucleosome assembly as well as disassembly yielding increased or reduced nucleosome density^57^. Importantly, each factor has the ability to open or shut chromatin accessibility^57^. Since ChIPs for Lsh demonstrates its presence at the Bmp4 gene, Lsh may directly augment chromatin access at these sites. In addition, the modest decrease in CG methylation at enhancer 2 of the *Bmp4* gene in *Lsh−/−* NSPCs may be a direct site-specific change due to Lsh deletion. Alternatively, the presence of DNA binding factors (transcriptional activators or repressors) could modulate the DNA methylation footprint^60^. Interestingly, many enhancers marked with H3K27ac can be highly DNA methylated^59^, as shown here for Bmp4 ECR2. Consistent with reduced chromatin accessibility at the *Bmp4* regulatory site, we observed a reduction of H3K4me1 modification, and mRNA and protein reduction due to Lsh deletion. These observations support the hypothesis that the *Bmp4* gene may serve as critical target for Lsh actions.

The ratio of H3K4me1 and H3K4me3 signifies enhancer^61^ and removal of H3K4me1 can accompany a switch from active to poised enhancers^62^. Interestingly, the reduction of H3K4me1 at the Bmp4 enhancer was not associated with a concomitant downregulation of H3K27ac, which occurs, for example, after depletion of MLL3 or MLL4^63, 64^. While we do not know the precise mechanism yet, it should be noted that active enhancers show varying levels of H3K4me1 and H3K27ac, and only about 80% of predicted enhancers (marked by H3K4me1 and H3K27ac) are active based on reporter assays^65, 66^. Also, we do not know if H3K4me1 and H3K27ac reside on the same nucleosome, or if additional changes occur that can influence the activity of enhancers, such as alterations of histone marks, H3K36me3 and H3K9me3, which distinguish active, intermediate and repressed states^66^, or changes of H4 acetylation or H3K4me2 present at enhancers^61^.

Bmp4 plays important roles in morphogenesis^51^, self-renewal of diverse stem cells^67, 68^, regulates the responsiveness to EGF^51^ and controls the transition between quiescence and proliferation in neural stem cells^49^. Cdkn1a, a cell cycle inhibitor, has been shown to inhibit the growth of adult and embryonic neuronal stem cells^46, 69^. Cdkn1a can directly engage at the Sox2 promoter region and suppresses expression of Sox2^47^, a factor important in maintaining neural stem cells^70^. Since we observed reduced Sox2 level in the absence of Lsh, a regulatory network controlled by Lsh, may involve Sox2 inhibition through enhanced Cdkn1a protein levels. While our data suggests a role for Bmp4 and Cdkn1a in the proliferation/survival of NSPCs, it does not exclude the contribution of other pathways. While we did not detect signs of genomic instability leading to p53 activation, DNA damage pathways can become activated in the absence of Lsh^69^, and may result in increased genomic instability and a decrease in survival. Alternatively, our RNA-seq analysis found several significantly de-regulated genes which may contribute to decreased growth/survival of *Lsh−/−* NSPCs; for example, the IPW gene, a long noncoding RNA which is overexpressed in the Prader-Willi syndrome, a genetic condition with multiple deficiencies, including cognitive and behavioral disturbances^71^, the cell cycle regulator Plk1, Cdca3 and the centromeric protein Cenpf, which can modulate proliferation and neurodevelopmental associated functions^72–74^, the transcription factors Dlx1 and Dlx2, which play a role in brain and craniofacial development^75^, and the Aspm genes that control brain size^76^. Thus several factors that are abnormally expressed in Lsh mutant NSPCs may potentially synergize and contribute to impaired neural stem cell renewal.

ICF patients suffer from a severe disorder, which includes immune malfunction and neurologic deficiencies. Here, we report for the first time, that Lsh ablation alters chromatin states at important regulatory genes and induces impaired self-renewal/growth of NSPCs, suggesting a direct role for Lsh in nervous system development. Revealing molecular pathways that cause developmental defects is critical in understanding the pathophysiology of neural syndromes and to advance future treatment options for cure.

## Materials and Methods

All procedures were carried out in accordance with regulation and guidelines of the National Cancer Institute, Center for Cancer Research, Frederick National Laboratory for Cancer Research, National Institutes of Health, Frederick, MD 21701. Animal use and housing was approved under protocol 13–276 by the “Animal Care and Use Committees, Frederick” of the NIH Office of Laboratory Animal Welfare. NCI-Frederick is accredited by AAALAC International and follows the Public Health Service Policy for the Care and Use of Laboratory Animals. Animal care was provided in accordance with the procedures outlined in the “Guide for Care and Use of Laboratory Animals (National Research Council; 1996; National Academy Press; Washington, DC).

### Tissue Dissociation and NSPCs Culture

Tissue dissociation and neurosphere generation were conducted using published protocols^77^. In short, at day 13.5 of gestation (plug date is considered at day 0.5) we dissected the whole cerebral neocortex of the embryos according to the methods established by Ahlenius *et. al*.^77^. Cerebral cortices were placed into 0.5 mL pre-warmed dissociation solution containing DMEM, 1 mM glutamine, 1 mM sodium pyruvate, 1 mM N-acetyl-cysteine (Sigma), and incubated with 10 unit/mL Papain (Worthington) for 20 min at 37 °C with 2–3 times of brief agitation. The samples were rinsed 3 times with dissociation solution and collected by gentle centrifugation. After the final wash, the tissue was mechanically dissociated into single-cell suspension by trituration 15–20 times in Neurobasal medium (Gibco) supplemented with 1mM L-glutamine, penicillin-streptomycin, B-27 serum-free supplement (Gibco) and N-2 supplement (Gibco), EGF (epidermal growth factor, 20 ng/mL) (Sigma) and bFGF (basic fibroblast growth factor; 10 ng/mL) (Gibco). Neurosphere cultures were treated with 10 ng/mL Bmp4 (R&D Systems) and 20 ng/mL Noggin (R&D Systems) as indicated at the time of plating. After cell number counting and viability measurement, cells were seeded into flat bottom ultra-low attachment multiple well plates (Thomas scientific). The cells will form floating neurospheres after overnight culture at 37 °C, 5% CO_2_. Neurospheres were passaged every 4–5 days by dissociation using 1 mL of TrypLE Express (Invitrogen) for 10 min, followed by 60–70 times pipetting to get single cells which were then cultured in fresh medium.

### Neurosphere Formation Assay

The neurosphere assay was conducted to determine the neural stem cells self-renewal capacity^32, 36^. In brief, mouse neurospheres were dissociated into single cell suspension, and reseeded at a density of around 5–10 cells per μL, in a 24-well flat bottom ultra-low attachment plates for clonal growth. The newly formed neurospheres were characterized 3 to 5 days afterward by GelCount (Oxford Optronix). The number and diameter of the spheres were analyzed using GelCount software under similar and optimized parameters with a minimum cutoff 20 μm in diameter. Cells from 3 to 5 embryos were analyzed for each assay. Every sample was repeated with 3–5 wells for each passage.

### NSPCs Differentiation Assay

NSPCs derived from E13.5 embryos or neurospheres were differentiated via two alternative methods: undirected differentiation and lineage oriented differentiation. For undirected differentiation, single cells dissociated from neurospheres were seeded at 20,000 cells/mL onto coverslips pre-coated with poly-D-lysine (100 ng/mL) and laminin (20 ug/mL). Cells grow in neurobasal medium supplemented with 1 mM L-glutamine, penicillin-streptomycin and 4% horse serum with no other growth factors. Cells were incubated in adhesion cultures for 5 days to allow differentiation into multiple linages in the population.

Alternatively, neurospheres were subjected to oriented differentiation protocols to promote differentiation into one dominant linage in the population (http://www.thermofisher.com/us/en/home/references/protocols/neurobiology/). To obtain neuron differentiation the dissociated neurospheres were cultured on dishes coated with poly-L-ornithine (20 ng/mL) and laminin (20 ug/mL) in neurobasal medium supplemented with 1 mM L-glutamine, penicillin-streptomycin, B-27 serum-free supplement and 0.5 mM dibutyryl (Sigma) for 7 days. For astrocyte differentiation, dissociated neurospheres were cultured in D-MDM supplemented with N-2, GlutaMAX and 1% FBS on Geltrex-coated dishes for 7 days. For oligodendrocyte differentiation, dissociated neurospheres were cultured in Neurobasal medium supplemented with 1 mM L-glutamine, penicillin-streptomycin, B-27 serum-free supplement and 30 ng/mL T3 (Sigma) on poly-L-ornithine (20 ng/mL) and laminin (20 ug/mL) coated dishes. For RT-qPCR results, the significance analyses were based on 3 technical repeats, shown are two biological replicates.

### Cell Proliferation Assay

Cell proliferation was measured by the Cell Proliferation BrdU (colorimetric) ELISA assay detection kit (Roche). NSPCs were dissociated into single cells and were seeded into 96 wells (5 × 1e4 cells per well). Cell culture medium was also incubated and performed as blank control. After 5 days culture, BrdU labeling reagent was added into the medium and continuously cultured for 20 hours, then ELISA were performed following the manufacturer’s instructions. Absorbance was read at 415 nm (reference wavelength 490 nm) on a microplate reader. BrdU incorporation value was expressed after background deduction.

Cell viability was measured by Cell Proliferation Reagent WST-1 (Roche) assay. NSPCs culture was similar to those used for BrdU ELISA assay. 10 μL per well of WST-1 was added 4 hours before measurement. Absorbance was read at 450 nm (reference wavelength 655 nm) on a microplate reader. Cell viability was presented as a value after the background deduction.

### Flow cytometry quantification

For the assays using neurospheres, the cultured neurospheres were first dissociated by TrypLE Express (Invitrogen) for 20 min, followed by 60–70 times pipetting to get single cells. For the differentiated linage cells assay, cells were detached by TrypLE Express, pipetted into single cells and suspended in PBS. Dissociated cells were fixed in 1% paraformaldehyde for 10 min, and the standard immunostaining protocol was performed. Cell suspensions were stained with antibodies on ice for 1 hour. The supernatant was aspirated, secondary antibody was added, and reactions were incubated in the dark on ice for 1 hour. Cells were rinsed and spun again as described. The cell suspension was passed through a nylon mesh to remove undigested fragments. The final cell pellet was suspended in 400 μL of flow buffer. FACS Calibur (BD Biosciences) instruments were used for analysis (Flow cytometry core facility of NCI/NIH). Results analysis was performed using FlowJo software.

### ChIP

The chromatin preparation and immunoprecipitation procedure is as described in ref. 78. An amount of 1 × 10e6 cells dissociated from neurospheres were digested by TrypLE Express (Invitrogen) for 40 min, followed by 60–70 times pipetting to get single cells, suspended in cold 1xPBS, and chemically cross-linked by addition of formaldehyde to a final concentration of 1% for 15 min at room temperature, then the cross-linking reaction was quenched by adding glycine to a 125 mM final concentration. We did not perform a significant test for the ChIP-qPCR assay since 5 to 6 independent derived samples from embryos of either genotype had to be pooled to obtain sufficient material. We repeated the experiment twice and found the results are reproducible. An amount of 4 μg antibodies anti-IgG, (anti-Lsh (custermized), anti-H3K4me1 (ab8895, Abcam), anti-H3K27ac (ab4729, Abcam)) were incubated with the sonication fragmented chromatin fragments. Input DNA was set aside and used as internal control. Student’s *t*-test was used for the enrichment significance test. qPCR primer sequences and their genomic locations are listed in Supplement Table 3.

### Nucleosome occupancy assay (NOMe-Seq)

Nucleosome occupancy assay was performed using NOMe-Seq kit (Active Motif) as described^16^. Nucleosome occupancy assay was performed using NOMe-Seq kit (Active Motif). Briefly, cells were treated with trypsin and centrifuged for 3 min at 500 g, then washed in ice-cold PBS and resuspended in 1 mL ice-cold nuclei buffer (10 mM Tris [pH 7.4], 10 mM NaCl, 3 mM MgCl_2_, 0.1 mM ethylenediaminetetraacetic acid (EDTA) and 0.5% NP-40, plus protease inhibitors) per 5 × 10e6 cells and incubated on ice for 10 min. Nuclei were recovered by centrifugation at 900 g for 3 min and washed in nuclei wash buffer (10 mM Tris, pH 7.4, 10 mM NaCl, 3 mM MgCl_2_ and 0.1 mM EDTA containing protease inhibitors). Freshly prepared nuclei (2 × 10e5 cells) were sonicated to generate fragments of more than 1 kb, then treated with 200 U of M.CviPI (NEB) in 15 μL 10x reaction buffer, 45 μL 1 M sucrose and 0.75 μL S-adenosyl methionine (SAM) in a volume of 150 μL. Reactions were quenched by the addition of an equal volume of Stop Solution (20 nM Tris-HCl, pH 7.9, 600 mM NaCl, 1% sodium dodecyl sulphate, 10 mM EDTA, 400 μg/mL Proteinase K) and incubated at 55 °C overnight. The chromatin was subjected to reversal crosslink, RNAase A and Protein K treatment then purified by phenol/chloroform extraction and ethanol precipitation.

Bisulfite conversion was performed using the MethylDetector kit (Active Motif). Primers and genomic locations information are listed in Supplement Table 3. PCR products were separated and cloned using the TA Kit (Qiagen), both according to the manufacturers’ instructions.

### RNA-seq data analysis and Differentially Expressed Genes (DEG) detection

Total RNA from NSPCs passage 2 was used for mRNA isolation and cDNA library construction with the TruSeq RNA Sample Preparation Kit (Illumina). Clusters were generated with the TruSeq PE Cluster Kit v3-HS & TruSeq SBS kit v3-HS according to the reagent preparation guide. The RNA sequencing was performed using the Illumina HiSeq2500. High quality 100 bp reads were aligned to the mouse reference genome (mm9) using TopHat 2.0.8^79^. The expression levels for each of the genes were normalized to reads per kb of exon model per million mapped reads (RPKM) to compared mRNA levels between samples. Differentially expressed genes were identified by Cuffdiff form Cufflinks 2.2.0 with default parameters^80^.

### Statistical Analysis

Quantification is shown of at least three independent experimental repeats. Student’s t-test was used to determine significance. All the tests were two-tailed. Error bars indicate standard deviation. A *p* -value below 0.05 was considered statistically significant. All cell-based assays include 3 to 5 biological replicates with 3 to 5 technical repeats for each sample. We performed the significance test using the entire set of data for each category.

## Electronic supplementary material

Supplement Information

Supplement Table 1

Supplement Table 2

Supplement Table 3
