# Supplementary material for: Lsh/HELLS regulates self-renewal/proliferation of neural stem/progenitor cells
Source: Sci Rep. 2017 Apr 25;7:1136. doi: 10.1038/s41598-017-00804-6 (PMC5430779; doi:10.1038/s41598-017-00804-6)

Supplemental Information

Lsh/HELLS regulates self-renewal/proliferation of neural stem/progenitor cells

Yixing Han[1], Jianke Ren[1], Eunice Lee[1], Xiaoping Xu[1], Weishi Yu[1] and Kathrin Muegge[1, 2, *]

[1]Mouse Cancer Genetics Program, Center for Cancer Research, National Cancer Institute, Frederick, Maryland 21702, USA

[2]Basic Science Program, Leidos Biomedical Research, Inc., Mouse Cancer Genetics Program, Frederick National Laboratory for Cancer Research, Frederick, Maryland 21702, USA

Running title: Lsh promotes neural stem/progenitor cells renewal

Supplemental Materials and Methods

Time-lapse imaging microscopy

Time-lapse imaging microscopy of primary NSPCs culture was performed with a cell observer (Zeiss) at constant conditions of 37°C and 5% CO_2. Grid glass bottom dishes were used for relocation of cells after immunofluorescence staining. Phase contrast images were acquired every 20 minutes for 25h using a 20x phase contrast objective (Zeiss). Images were analyzed using Image J software (National Institute of Health, USA).

qPCR assay

Total RNA was extracted from neurospheres or differentiated NSPCs using RNeasy Mini Kit (Qiagen) with DNase treatment and oligo-d (T) primed reverse transcription was executed using the Superscript III kit (Invitrogen). SYBR Green Dye real-time qPCR was used for the quantitative PCR reaction at Bio-Ras MyiQ2 system. Expression level was first normalized to internal GAPDH abundance, and then relative to mRNA level to WT1 in each condition. Student's *t*-test was used for the differentially expression level significance test. Primers and genomic locations information are listed in Supplement Table 2.

TUNEL assay

Apoptosis levels were detected in embryonic brain frozen sections using the terminal deoxynucleotidyl- transferase-mediated dUTP-biotin nick end-labeling (TUNEL) assay (ApopTag Red In Situ Apoptosis Detection Kit, S7165, Millipore) following the manufacturer's instruction. Images were acquired using a Zeiss microscope. Positive

signals from multiple sections were quantified by Image J software by picking random 5 - 10 SVZ fields with the same magnification. Positivity was calculated as the average percentage of total cells.

Flow cytometry quantification

For the assays using neurospheres, the cultured neurospheres were first dissociated by TrypLE Express (Invitrogen) for 20 min, followed by 60-70 times pipetting to get single cells. For the differentiated linage cells assay, cells were detached by TrypLE Express, pipetted into single cells and suspended in PBS. Dissociated cells were fixed in 1% paraformaldehyde for 10 min, and the standard immunostaining protocol was performed. Cell suspensions were stained with antibodies on ice for 1 hour. The supernatant was aspirated, secondary antibody was added, and reactions were incubated in the dark on ice for 1 hour. Cells were rinsed and spun again. The cell suspension was passed through a nylon mesh to remove undigested fragments. The final cell pellet was suspended in 400 μL of flow buffer. FACS Calibur (BD Biosciences) instruments were used for analysis (Flow cytometry core facility of NCI/NIH). Result analysis was performed using FlowJo software.

Immunofluorescence staining

Adherent mouse NSPCs were fixed in 4% PFA for 10 min at room temperature (25°C), rinsed twice with PBS and then 0.5% Triton X-100 in PBS for 10 minutes at RT and then blocked with 5% bovine serum albumin (BSA) for 30 minutes at RT. Cells were then incubated with primary antibodies in blocking solution overnight at 4°C. The following primary antibodies were used to detect intracellular antigens: mouse monoclonal anti-

Nestin (1:200, ab6142, Abcam), and rabbit polyclonal anti-survivin (1:400, Cell Signaling Technology), anti-Numb (1:1000, ab14140, Abcam), anti- Vimentin (1:200, ab7260, Abcam), anti-Tuj1 (1:150, ab15568, Abcam), anti-GFAP (1:500, ab7260, Abcam), anti-O4 (1:250, MAB1326, Millipore). After washing in PBS, cells were incubated in secondary antibodies [DyLightTM 594-conjugated goat anti-(rabbit IgG) and DyLightTM 488-conjugated goat anti-(mouse IgG); 1:200 dilution] at room temperature for 1h and the nuclei were counterstained with DAPI (4, 6-diamidino- 2-phenylindole). Images were captured with a fluorescence microscope (Zeiss) and processed with Image J software.

For embryonic brain staining, dissected brains were fixed in 4% PFA at 4°C overnight, cryopreserved with 15% and 30% sucrose in PBS sequentially, bedded in OCT (optimal cutting temperature) compound and cryostat sectioned (10μm) on to slides. The slides were stained as described above.

Immunohistochemistry (IHC)

IHC procedure is following previous publications[1]. Embryonic mouse brain tissue were dissected at indicated ages and fixed overnight in 10% neutral buffered formalin, transferred to 70% ethanol, routinely processed, and embedded in paraffin. Tissues were sectioned for 10 successive layers at 10μm intervals and stained with haematoxylin and eosin (H&E) for histopathological examination. Antibodies included: anti-Lsh (1:1000, rabbit polyclonal), anti-Bmp4 (1:1000, ab39973, Abcam), anti-Cdkn1a (1:100, rabbit polyclonal ab2961, Abcam), anti-Ki67 (1:200, ab15880, Abcam), anti-Sox2 (1:500, ab97959, Abcam). The first primary antibody was incubated overnight followed by the second primary antibody (goat anti-rabbit) incubation for 2 hours at room temperature. The

peroxidase and substrate reaction follows the antibody incubation and washing (NovaRED Peroxidase (HRP) Substrate Kit, SK-4800, Vector), DAPI as the counterstaining, following washing, the sections were mounted under coverslips using Vectashield with DAPI (ProLong® Diamond Antifade Mountant with DAPI, P36962, Life Technology).

References

1. Song, Y., et al., Carcinoma initiation via RB tumor suppressor inactivation: a versatile approach to epithelial subtype-dependent cancer initiation in diverse tissues. PLoS One, 2013. 8(12): p. e80459.

Titles and legends of supplement figures

Supplement Figure 1. Asymmetric cell divisions in *Lsh-/-* NSPCs are not affected. A and B. Representative IF images from time-lapse microscope on differentiated NSPCs using asymmetric division marker anti-Numb and neuron progenitor markers anti-phVimentin (A) and anti-Survivin (B). C. Summary of asymmetric cell division proportions after time-lapse imaging and IF staining. The data in C57BL/6 was set as the baseline.

Supplement Figure 2. Lineage gene expression in *Lsh-/-* NSPCs and assessment of p53 activation in NSPCs self-renewal. A and B. RT-qPCR confirmation of the gene expression level change in NSPCs proliferation: Tuj1 (A) and Mbp (B). C and D. p53 mRNA level (C), protein level and phosphorylation level (D) are comparable in WT and *Lsh-/-* NSPCs. Wild-type ES cells were treated by 250 mM methylsulfonylmethane (MMS) for 48 hours, and served as positive control. Data are represented as mean ± SD. Student's *t*-test was used to determine significance. * $p<0.05$, ** $p<0.001$. N.S. is not significant.

Supplement Figure 3. Gene Ontology (GO) analysis of differentially expressed genes. GO results from DAVID on the de-repressed (A), up- (B) and down-regulated (C) genes in *Lsh-/-* NSPCs. X-axis represents negative log *p*-values.

Supplement Figure 4. Effect of Bmp4 and Noggin on growth/survival in WT and *Lsh-/-* neurospheres. A and B. Quantification of neurosphere numbers (A) and average diameters (B) of WT and *Lsh-/-* NSPCs upon a moderate amount (10 ng/mL) of exogenous Bmp4 addition. The numbers have been normalized to the WT cultured under normal

conditions. C and D. Quantification of the neurosphere numbers (C) and average diameters (D) of WT and *Lsh-/-* NSPCs upon a low amount (2 ng/mL) of exogenous Bmp4 addition. Noggin is used as antagonist of Bmp4 to reverse/block Bmp4 effects (3 WT and 5 KO embryos were used, 3 technical repeats for each sample). Error bars represent SD. * p<0.05, ** p<0.001.

Supplement Figure 5. Cdkn1a transcription level in *Lsh-/-* NSPCs upon Bmp4 treatment. RT-qPCR assay for detection of *Cdkn1a* mRNA level changes upon Bmp4 addition. EGF and FGF2 are necessary growth factors used for the growth of NSPCs *in vitro* . Noggin (20 ng/mL) is used as antagonist of Bmp4 to reverse/block Bmp4 (10 ng/mL) effects. Three samples derived from three individual embryos were used as biological replicates. Data are represented as mean ± SD. Student's *t*-test was used to determine significance.

Supplement Figure 6. Lsh presence at enhancer sites of Bmp4 and Cdkn1a genes. ChIP-qPCR assay for detection of Lsh association at Bmp4 enhancer regions and Cdkn1a enhancer regions as depicted in Figure 4 comparing wild type (WT) and *Lsh-/-* samples. Oligo2 represent positive and NC 11,17,18,19,22 represent negative controls (four intergenic and one intron region, Supplement Table 3). Of note is a minor precipitate in *Lsh-/-* samples which may be due to a small amount of mutant Lsh protein accumulating at chromatin; alternatively, the Lsh-antiserum may show slight non-specific cross-reactivity. Input DNA for ChIP assay served as internal amplification control. Data are represented as mean ± SD.

Supplemental Figure 7. DNA methylation changes at *Bmp4* and *Cdkn1a* enhancer regions. Complementation to Figure 4C. A. CpG methylation assessment by bisulfate sequencing assay on enhancer regions of *Bmp4*. B. CpG methylation assessment by bisulfate sequencing assay on enhancer regions of *Cdkn1a*. White circles represent unmethylated CpG sites, and black circles represent methylated CpG sites. WT and KO samples for BS assay n = 2.

Supplemental Figure 8. Altered nucleosome occupancy at the regulatory regions of *Bmp4* and *Cdkn1a*. Complementation to Figure 5. GpC methylation and nucleosome position assay by NOMe-seq at Bmp4 enhancer region (A) and Cdkn1a enhancer regions (B). WT and KO samples for NOMe-seq assay n =2.

Titles of supplemental tables

Table 1. Differentially Expressed Genes in Lsh deleted NSPCs identified by Cuffdiff from Cufflinks 2.2.0 with default parameters.

Table 2. Partek results of RNA-Seq analysis.

Table 3. Primer Sequences used in RT-qPCR, ChIP-qPCR, and BS-seq.

Supplement Figure 1

A

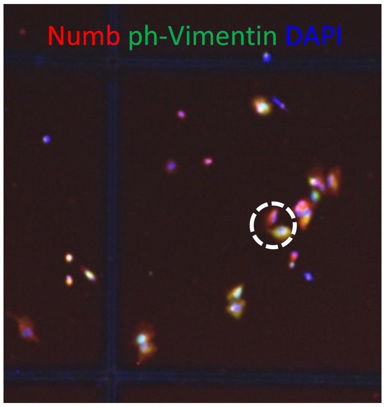

B

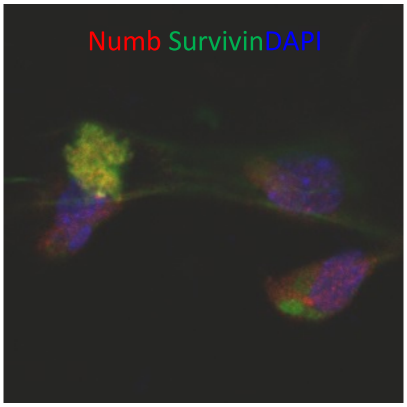

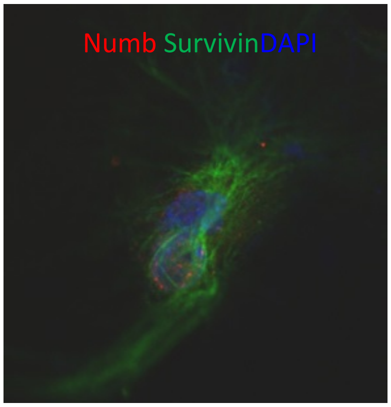

C

GG309	Pairs	ACD	Percentage
WT	161	51	31.68%
KO	373	128	34.32%

C57BL/B6	Pairs	ACD	Percentage
WT	95	32	33.68%

Supplement Figure 2

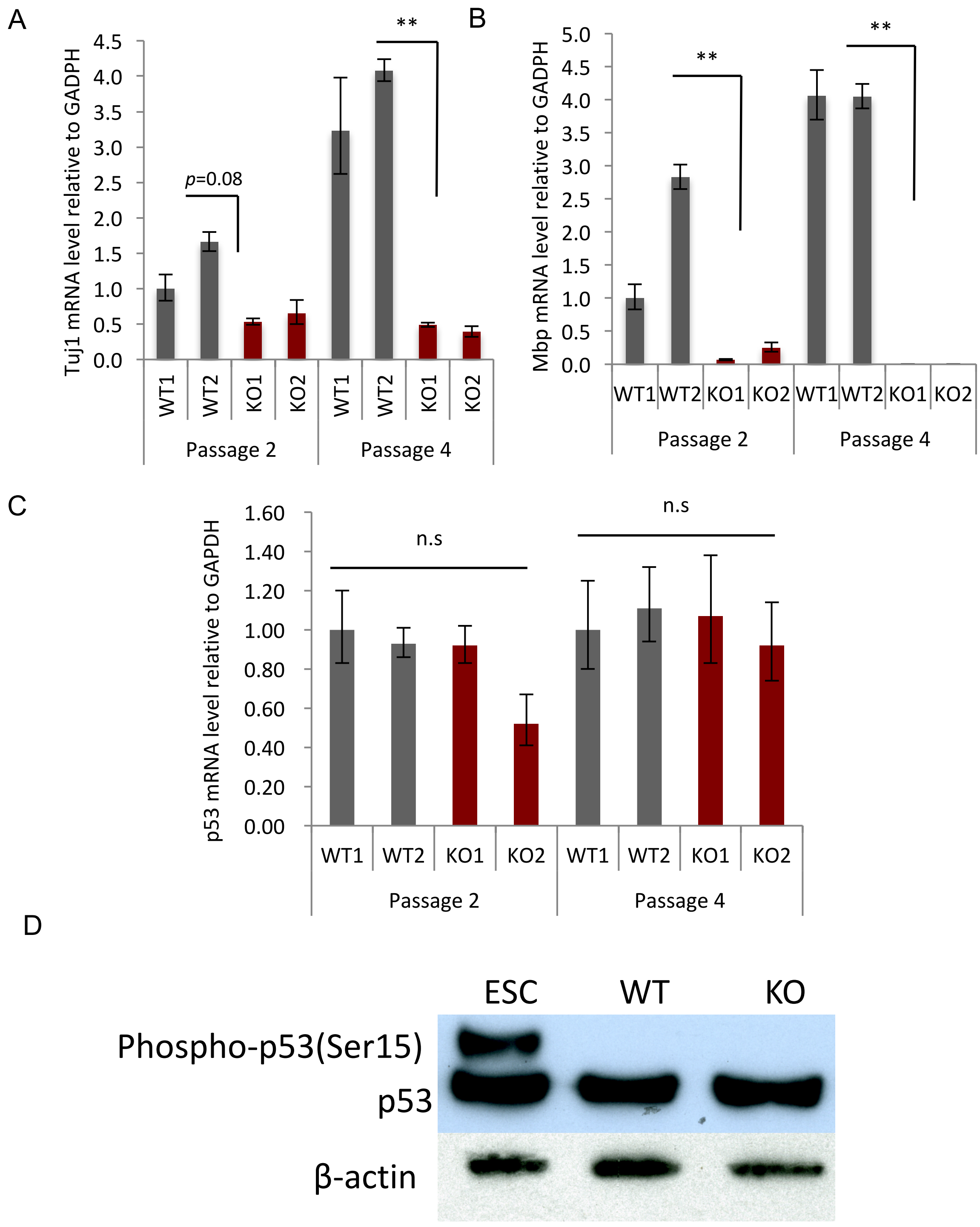

Supplement Figure 3

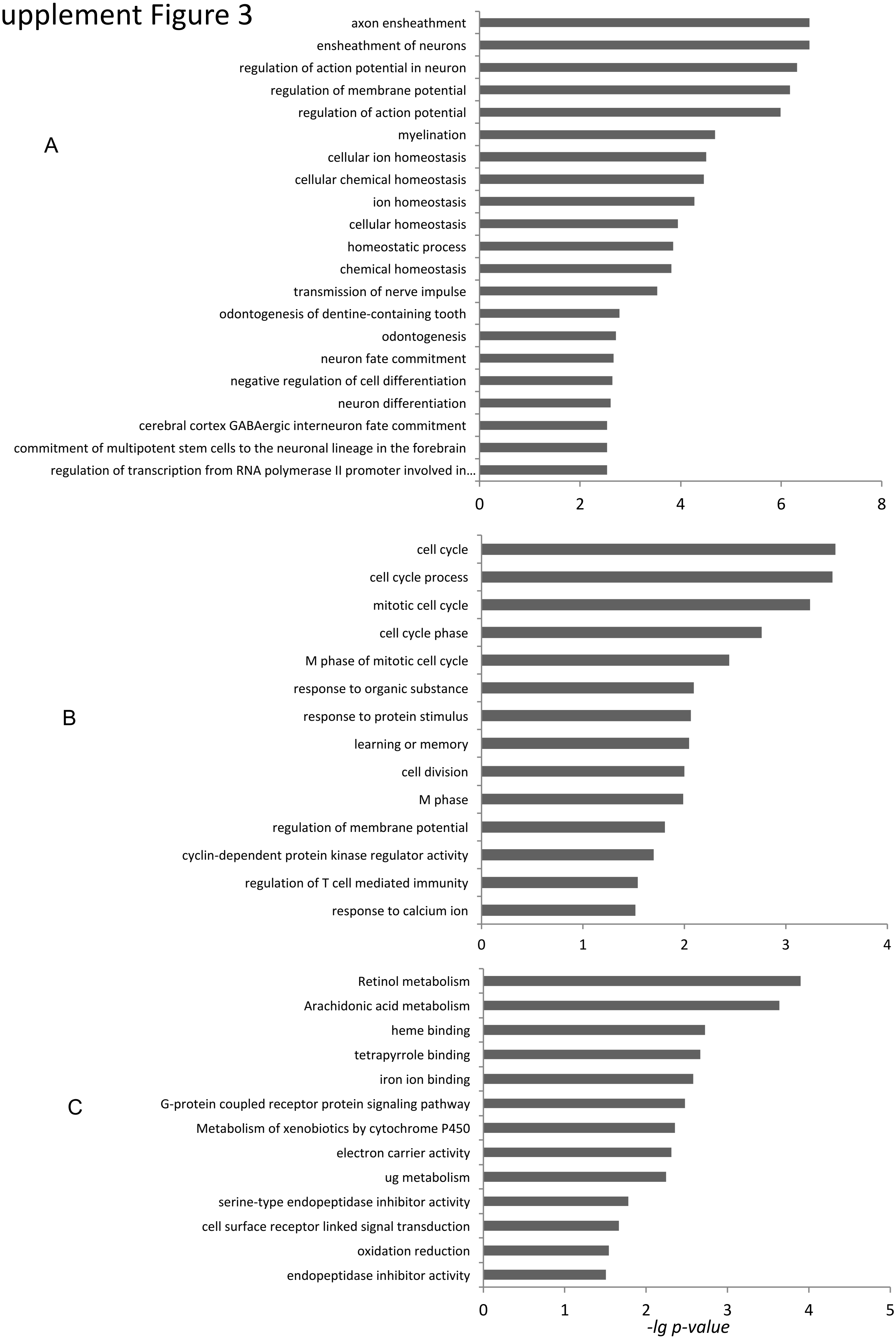

Supplement Figure 4

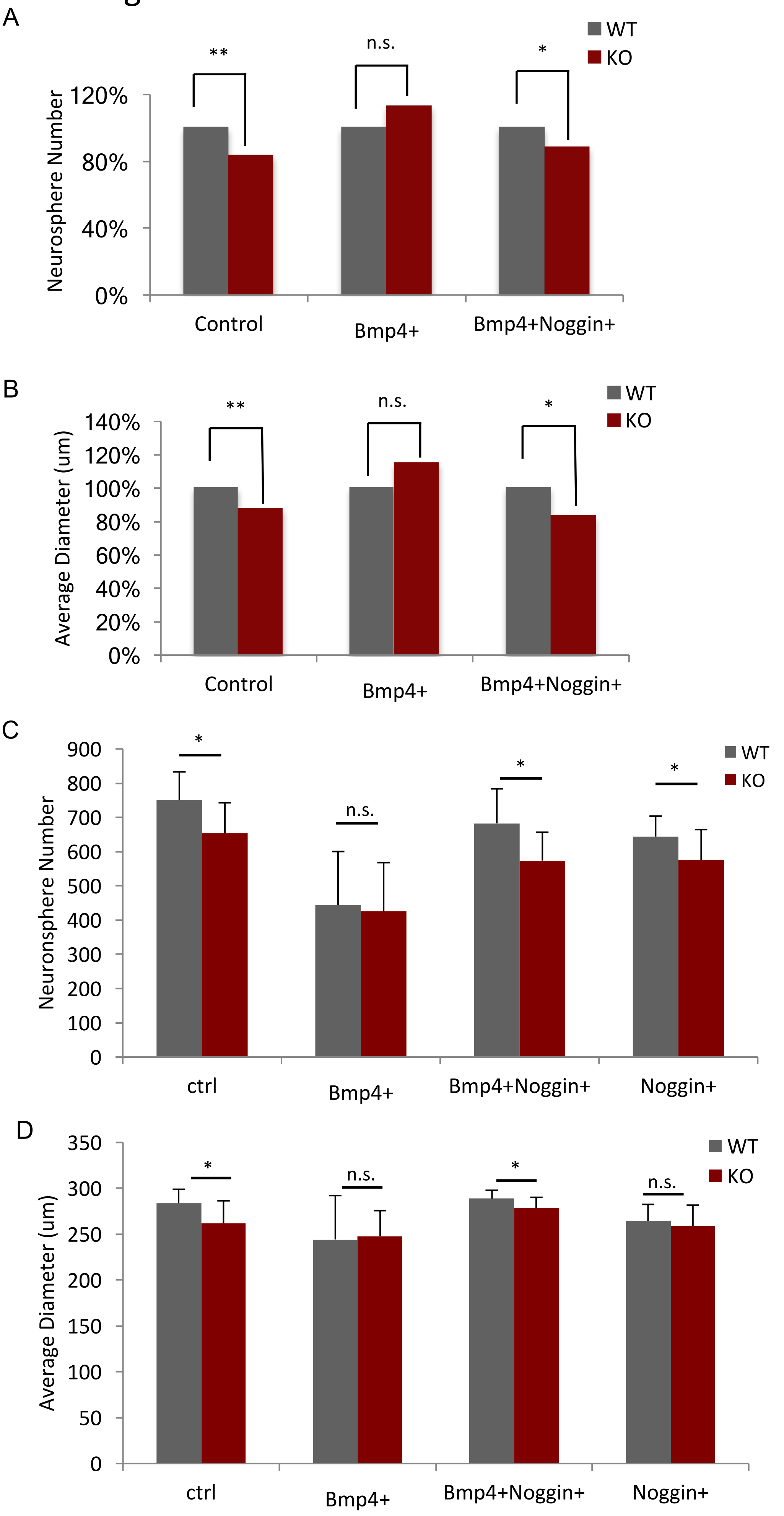

Supplement Figure 5

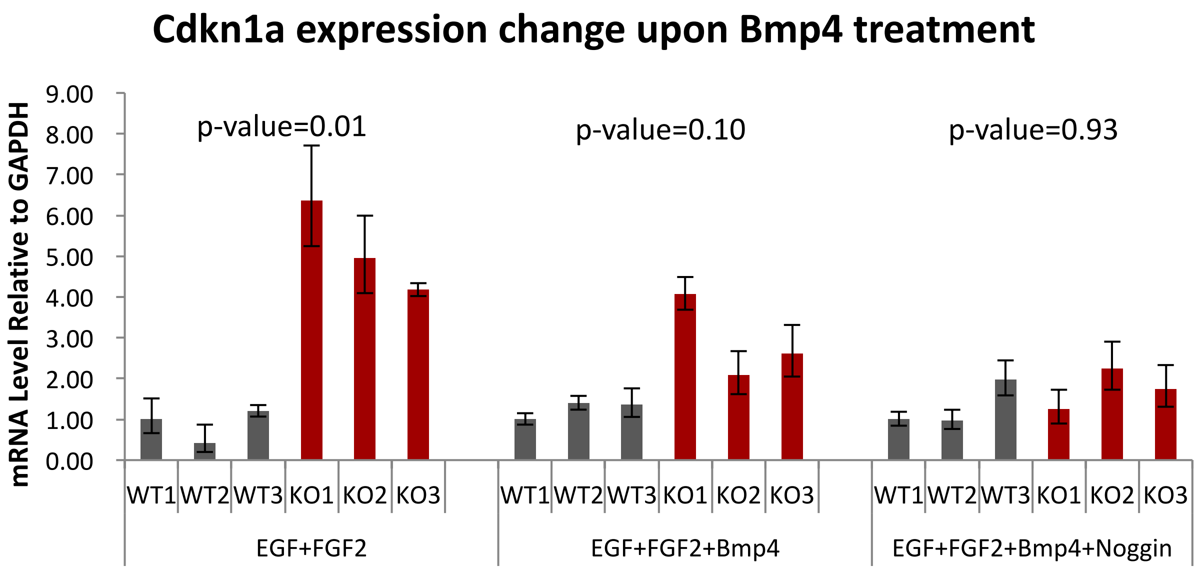

Supplement Figure 6

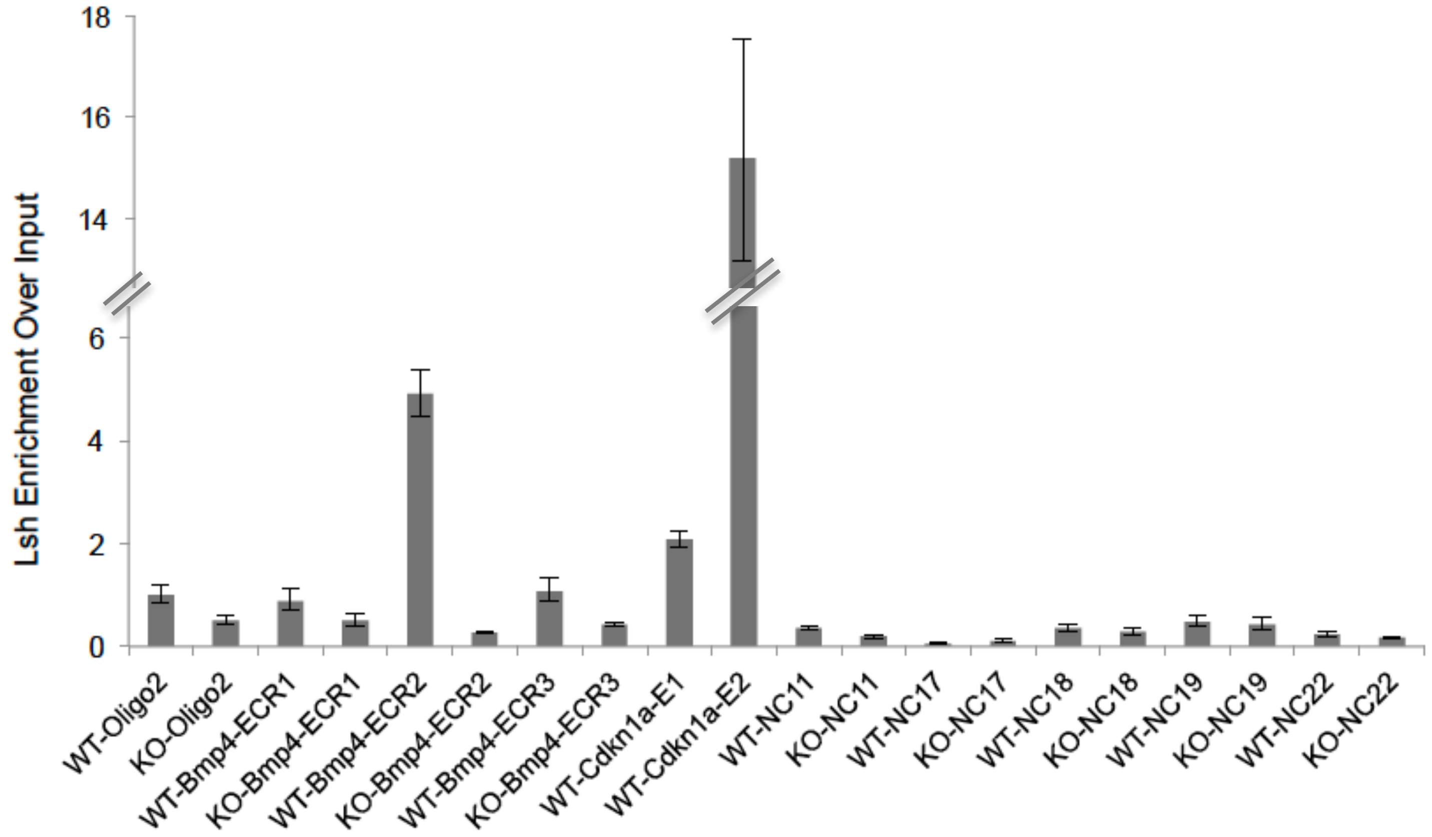

Supplement Figure 7

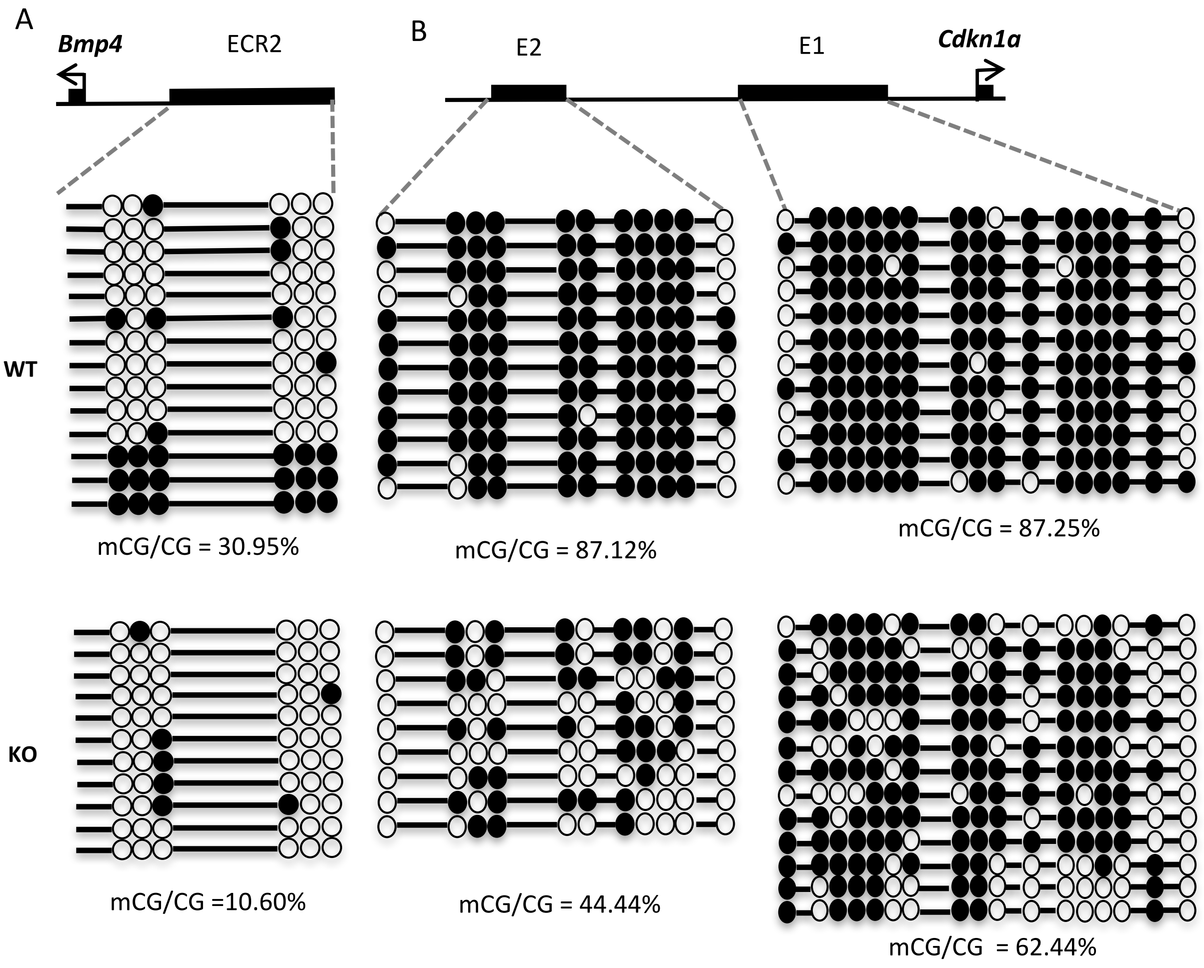

Supplement Figure 8

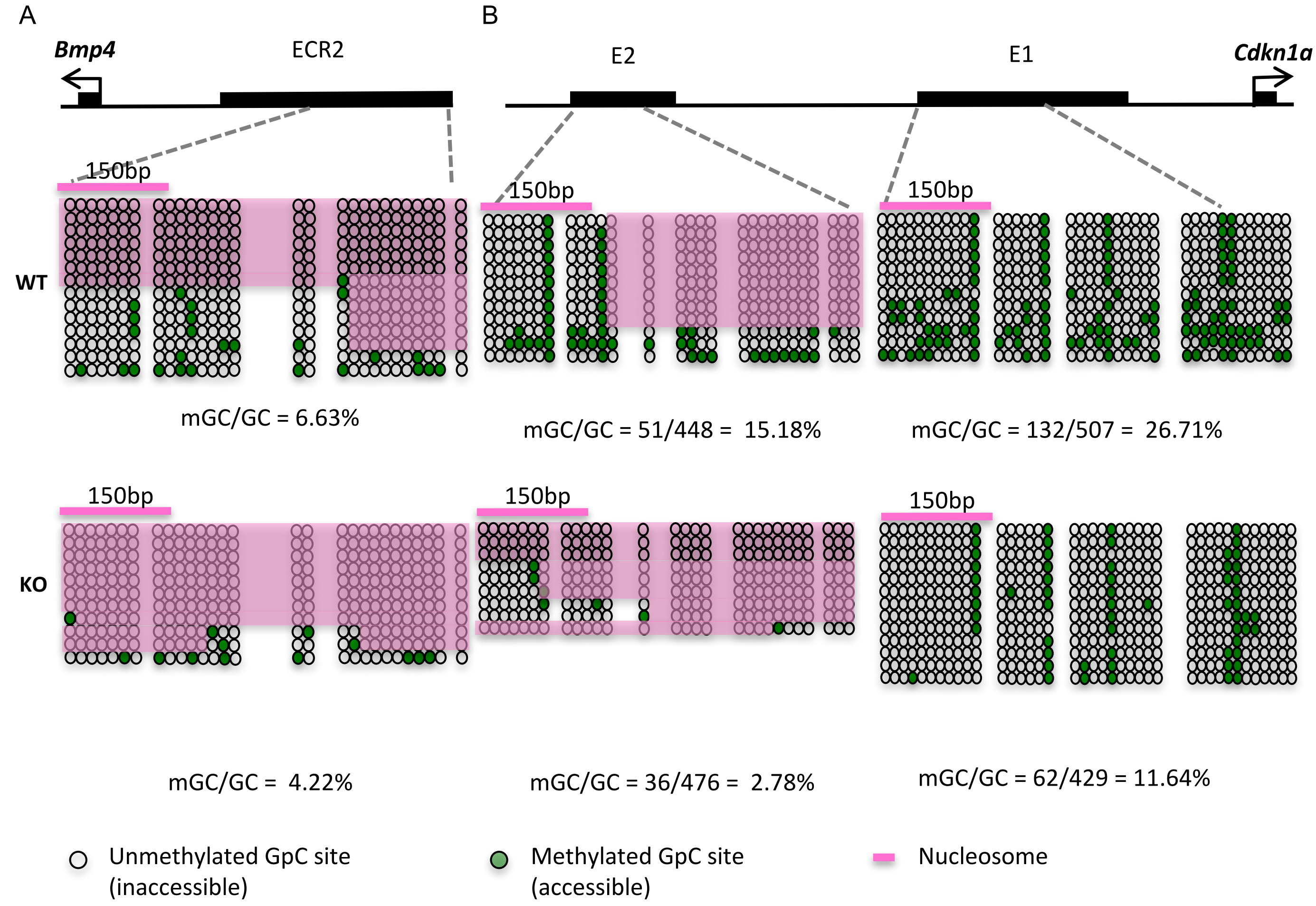

Supplement: Supplementary file 1 — Supplement Information [file 41598_2017_804_MOESM1_ESM.pdf]
